# The relationship between twitch depression and twitch fade during neuromuscular block produced by vecuronium: correlation with the release of acetylcholine

**DOI:** 10.1186/1742-4682-4-24

**Published:** 2007-07-16

**Authors:** Shashi B Bhatt, Jack Kohl, Anton Amann, Vladimir Nigrovic

**Affiliations:** 1 Department of Anesthesiology, College of Medicine, University of Toledo, Toledo, OH, USA; 2 Department of Anesthesiology and Critical Care Medicine, Medical University of Innsbruck, Innsbruck, Austria, and Department of Environmental Sciences, Federal Institute of Technology, Zürich, Switzerland

## Abstract

**Background:**

Train-of-four stimulation pattern following the administration of non-depolarizing neuromuscular blocking drugs reveals fade on successive contractions. Fade is caused by the release of fewer acetylcholine molecules by the fourth (A_4_) than by the first stimulus (A_1_). The current study was conducted to define the relationship between the clinically observed fade and the simulated decline in acetylcholine release (A_4_/A_1_) that would be necessary to produce it.

**Methods:**

The T_4_/T_1 _ratios produced by different doses of vecuronium (15–80 μg·kg^-1^) were plotted as a function of the concomitant T_1_. Separately in a model of neuromuscular transmission, T_1_, T_4_, and T_4_/T_1 _were estimated using simulations in the presence and in the absence of a neuromuscular blocking drug and a stepwise decrease in A_4_, but constant A_1_.

**Results:**

Vecuronium induced neuromuscular block was associated with larger T_4_/T_1 _ratios (less fade) during the onset than during the offset of the block. All doses caused similar fade during offset. Simulations revealed that the smallest T_4_/T_1 _was associated with the nadir of A_4_/A_1 _and occurred at the beginning of T_1 _recovery. The nadir of A_4_/A_1 _was only marginally affected by the dose of vecuronium: 15 μg·kg^-1 ^producing the minimum A_4_/A_1 _of 0.8 and 80 μg·kg^-1 ^the minimum A_4_/A_1 _of 0.7.

**Conclusion:**

The hysteresis in the fade between onset and offset appears to be caused by a delayed decrease of A_4_/A_1 _as compared with the decrease in T_1_. Tentative estimates of the decrease in A_4_/A_1 _during fade produced by vecuronium are offered. However, the validity of these estimates is dependent on the validity of the assumptions made in simulations.

## Background

Non-depolarizing neuromuscular blocking drugs cause a dose-dependent decrease in the indirectly evoked muscle contractions (twitches). Upon repetitive stimulation, the successive twitches during partial nondepolarizing block are reduced more than the first twitch. This phenomenon is known as fade. The ratio of the twitch strength produced by the fourth stimulus (T_4_) compared to that produced by the first stimulus (T_1_), i.e. T_4_/T_1_, elicited by a train-of-four (TOF) stimuli is of interest to both anesthesiologists and physiologists. Anesthesiologists use the T_4_/T_1 _ratio as an indirect measure of the still present muscle paralysis [1-3], while physiologists study the fade to gain insight into the processes of neuromuscular transmission [4-6].

Bowman [7] and others [4,8-10] have suggested that neuromuscular blocking drugs produce twitch fade during repetitive stimulation by binding to the presynaptic cholinoceptors and inhibiting the positive auto-feedback action of acetylcholine (ACh) on its own release elicited during repetitive stimulation. This action of neuromuscular blocking drugs causes the stimuli following the first stimulus to release fewer ACh molecules.

We have conducted the following study in an attempt to relate the clinically observed twitch fade, to the decrease in ACh that could account for the fade elicited by a train of four stimuli. In the first part of the investigation, we studied twitch depression and twitch fade in human subjects following the administration of different doses of vecuronium. In the second part, we expanded our model of neuromuscular transmission[11] by considering a decrease in the release of ACh elicited by the fourth stimulus.

## Methods

### Clinical experiments

After approval by the local IRB, 21 ASA physical status I or II, adult non-obese subjects scheduled for elective surgery consented to participate in the study. Following the i.v. administration of midazolam 1 – 2 mg and fentanyl 3 – 5 μg·kg^-1^, anesthesia was induced with propofol 2 – 3 mg· kg^-1^. Tracheal intubation was performed following the topical application of lidocaine (4%, 3 ml). Anesthesia was maintained with propofol infusion (100–150 μg·kg^-1·^min^-1^) and 66% N_2_O in oxygen. Ventilation was controlled to maintain normocapnia.

The thumb of one hand was abducted (preload between 250 g and 300 g) and connected to a force transducer (Gould FT-10, Grass Telefactor, West Warwick, RI). The signal from the force transducer was amplified, digitized at 100 Hz, and recorded on a PC using PolyVIEW data acquisition and analysis software (version 2.5, Astro-Med, Inc., West Warwick, RI) for subsequent analysis.

Supramaximal stimuli were delivered to the ulnar nerve at the wrist *via *skin electrodes. Baseline stabilization was obtained by applying 1 Hz stimuli for 20 minutes. Following the stabilization period, the stimulation was changed to a TOF pattern, i.e. four supramaximal stimuli at 2 Hz, and the trains repeated at 12 s intervals. Vecuronium was injected in doses ranging from 15 μg·kg^-1 ^to 80 μg·kg^-1^. The doses were chosen to produce a wide range of twitch depression, including the complete ablation of T_1_. The recorded strengths of T_1 _and T_4 _were expressed as fractions of T_1 _before the administration of vecuronium. The recordings were continued until complete spontaneous recovery from the neuromuscular block. The results are reported only for those subjects in whom T_1 _returned to within ± 10% of the baseline.

### Simulation of T_1 _and T_4 _as related to the release of acetylcholine by the first and the fourth stimuli

The strength of the twitches elicited by the first and the fourth stimuli in a TOF stimulation pattern was simulated in the model of neuromuscular transmission [11]. The model considers the stimulus-induced release of ACh into a synaptic cleft, ACh binding to two sites at a single postsynaptic receptor, and ACh hydrolysis. The number of ACh molecules released by the first stimulus was postulated to establish a constant initial concentration of ACh, [A]_1 _= 7.75 × 10^-6 ^M. The model was expanded by assuming that the fourth stimulus releases either the same or a smaller number of ACh molecules, and establishes the initial concentration of ACh in the synaptic cleft denoted by [A]_4_. The ratio [A]_4_/[A]_1 _was assigned nine values from 1.0 to 0.8 in steps of 0.025, and two additional values for [A]_4_/[A]_1 _= 0.75 and 0.70. The concentration of the postsynaptic receptors in the synaptic cleft, [R], was assumed to be 7.75 × 10^-5 ^M [11].

Binding of ACh to two sites at a postsynaptic receptor was defined by the association rate constant *k*_association _identical for both sites, *k*_association _= 1.35 × 10^8 ^M^-1· ^s^-1^. The assigned dissociation rate constants, *k*_dissociation_, were different: for site_1 _*k*_dissociation _= 1350 s^-1^, and for site_2 _*k*_dissociation _= 13500 s^-1^. The assignments define two equilibrium dissociation constants, *K*_A1 _= 10^-5 ^M for site_1_, and *K*_A2 _= 10^-4 ^M for site_2_. Affinities of ACh for the binding sites are the reciprocals of the equilibrium dissociation constants. After the release of ACh, three complexes are formed between ACh and the receptors: ARO, ORA, and ARA. Here, the first and the third letter indicate the species occupying site_1 _and site_2_, A is ACh, O is an empty site, and R is the postsynaptic receptor. The complex ARA represents the activated postsynaptic receptors. The concentrations of all three complexes increase transiently after the release of ACh, reach a peak, and subsequently decline due to hydrolysis of free ACh. We assigned a first-order rate constant for hydrolysis of ACh (*k*_hydrolysis_) at 12000 s^-1 ^[11].

The strength of T_1 _or T_4 _was calculated based on the assumption that a muscle fiber contracts when [ARA] reaches or surpasses the threshold level of [ARA] for that fiber [11]. The strength of T_1 _or T_4 _depends on the number of contracting fibers. All those fibers with a threshold [ARA] at or below the calculated peak value of [ARA] contract. The twitches, be it T_1 _or T_4_, were calculated identically using the calculated peak value of [ARA] after the first or the fourth stimulus:

Twitch=peak[ARA]γApeak[ARA]γA+[ARA50]γA
 MathType@MTEF@5@5@+=feaafiart1ev1aaatCvAUfKttLearuWrP9MDH5MBPbIqV92AaeXatLxBI9gBaebbnrfifHhDYfgasaacH8akY=wiFfYdH8Gipec8Eeeu0xXdbba9frFj0=OqFfea0dXdd9vqai=hGuQ8kuc9pgc9s8qqaq=dirpe0xb9q8qiLsFr0=vr0=vr0dc8meaabaqaciaacaGaaeqabaqabeGadaaakeaacqqGubavcqqG3bWDcqqGPbqAcqqG0baDcqqGJbWycqqGObaAcqGH9aqpdaWcaaqaaiabbchaWjabbwgaLjabbggaHjabbUgaRjabcUfaBjabbgeabjabbkfasjabbgeabjabc2faDnaaCaaaleqabaacciGae83SdC2aaSbaaWqaaiabbgeabbqabaaaaaGcbaGaeeiCaaNaeeyzauMaeeyyaeMaee4AaSMaei4waSLaeeyqaeKaeeOuaiLaeeyqaeKaeiyxa01aaWbaaSqabeaacqWFZoWzdaWgaaadbaGaeeyqaeeabeaaaaGccqGHRaWkcqGGBbWwcqqGbbqqcqqGsbGucqqGbbqqcqaI1aqncqaIWaamcqGGDbqxdaahaaWcbeqaaiab=n7aNnaaBaaameaacqqGbbqqaeqaaaaaaaaaaa@5DBC@

where γ_A _characterizes the distribution of the threshold [ARA] among the fibers of the muscle and [ARA50] is the median of all threshold [ARA] [11]. We used the previously defined values: γ_A _= 4.732 and [ARA50] = 9.524 × 10^-9 ^M [11].

The hypothetical muscle relaxant D competes with ACh for binding to the same two sites at a postsynaptic receptor. The assigned constants for the interaction were: *k*_associationD _identical for both binding sites, *k*_associationD _= 4.0 × 10^8 ^M^-1 ^s^-1^, and the equilibrium dissociation constants *K*_D1 _= 10^-7 ^for site_1 _and *K*_D2 _= 10^-6 ^M for site_2_. The dissociation rate constants were calculated from *k*_dissociationD _= *K*_D_·*k*_associationD_. Similar to ACh, the muscle relaxant D produces three types of complexes by itself, e.g. DRO, ORD and DRD, and two additional mixed complexes with ACh, ARD and DRA. The molar concentrations of D in the synaptic cleft are labeled [D]_synaptic cleft_. Higher [D]_synaptic cleft _leads to a larger fraction of the postsynaptic receptors occupied by D. In the calculations, we used 100 logarithmically equidistant values for a 10-fold increase in [D]_synaptic cleft _to produce T_1 _declining from its maximal value to 0.05. This was achieved by increasing [D] from 1· 10^-8 ^to 5· 10^6 ^M. We assumed that the decrease in T_1 _is produced exclusively by increased [D] and, hence, an increased occupancy of the postsynaptic receptors. On the other hand, the decrease in T_4 _is caused by a combination of increased postsynaptic receptor occupancy and the diminished release of ACh elicited by the fourth stimulus. Neuromuscular block (NMB) was defined as the difference in strength of T_1 _in the absence and the presence of a muscle relaxant.

The calculations were performed using the program MATHEMATICA (version 5.2) from Wolfram Research, Inc., Champaign, IL.

## Results

Although 21 subjects were studied, the first twitch returned to within ± 10% of the baseline in only 15 subjects and only the data from these subjects are presented. Since our measurement technique did not allow us to measure twitch tension with precision once T_1 _decreased to below 10% of its baseline value, we have not included in our analysis any data, be it for T_1 _or T_4_/T_1 _ratios, for T_1 _< 0.1.

**Figure 1:**
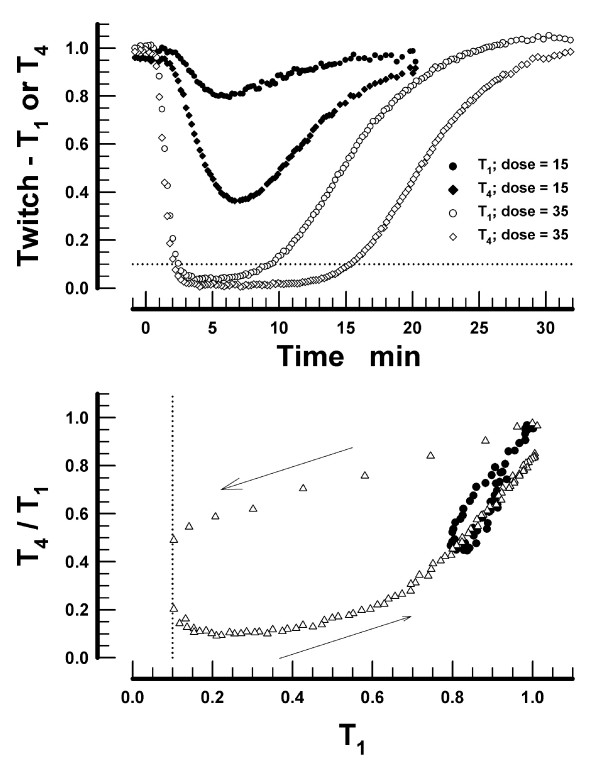
Time-course of the first (T_1_) and the fourth twitch (T_4_) in two subjects following the injection of vecuronium. The dose of vecuronium was 15 μg·kg^-1 ^in one subject (filled symbols) and 35 μg·kg^-1 ^in the second subject (open symbols, upper panel). The dotted line parallel with the Time axis indicates twitch = 0.1. The lower panel presents the T_4_/T_1 _ratios as a function of T_1 _for the same two subjects. The loops commence at T_1 _= 1.0 and T_4_/T_1 _= 1.0 and then follow the direction indicated by arrows. The dotted line parallel with the T_4_/T_1 _axis indicates T_1 _= 0.1.

In all subjects studied, the peak depression of T_4 _lagged behind the peak depression of T_1_, and the minimum T_4 _was always lower than the minimum T_1_. This is illustrated for two subjects who received vecuronium doses of 15 μg·kg^-1 ^or 35 μg·kg^-1^, respectively (upper panel in Figure 1). The plot of T_4_/T_1 _as a function of T_1 _for the same two subjects reveals that, for any given value of T_1_, the T_4_/T_1 _ratio is higher during the onset than during the offset of NMB (lower panel in Figure 1).

**Figure 2:**
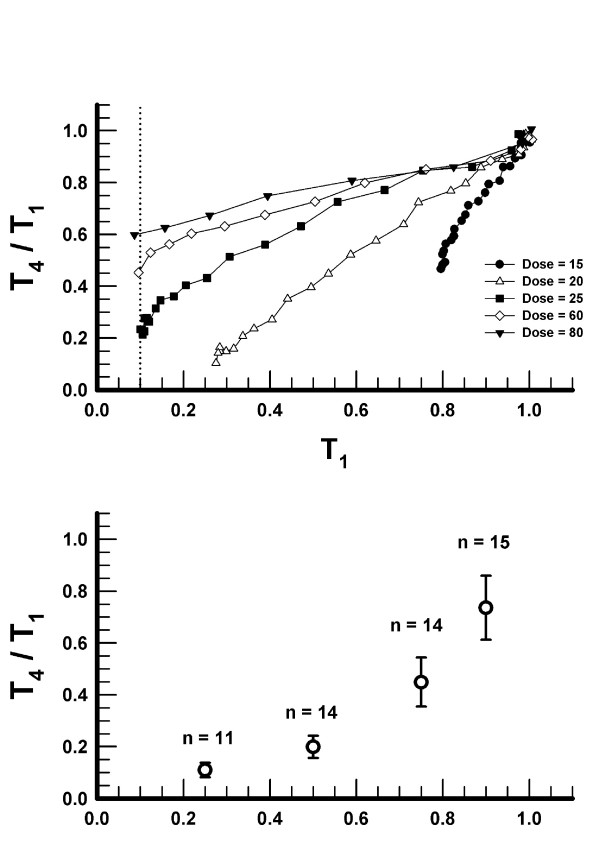
*Upper panel: *The ratios of the fourth to the first twitch (T_4_/T_1_) as a function of the first twitch (T_1_) for various doses (μg·kg^-1^) of vecuronium (individual subjects) during the onset of neuromuscular block. At the moment of vecuronium injection, T_1 _= 1.0 and T_4_/T_1 _= 1.0. *Lower panel: *T_4_/T_1 _ratio (mean ± SD) as a function of T_1 _(at T_1 _= 0.25, 0.5, 0.75, and 0.9) for all doses of vecuronium during the offset of neuromuscular block. The number of subjects is indicated by *n*. The temporal sequence of observations starts at the lowest value of T_1 _and progresses toward higher values of T_1_. The label for the X-axis is identical to the label of the X-axis in the upper panel.

The T_4_/T_1 _ratios as a function of T_1 _for various doses of vecuronium during the onset of NMB are presented in the upper panel of Figure 2. To simplify the presentation, only one representative subject is presented for each dose. The results indicate that, at any level of T_1_, the T_4_/T_1 _ratios are higher, i.e. the twitch fade is less pronounced, with larger doses of vecuronium. Thus, at T_1 _= 0.5, a vecuronium dose of 20 μg·kg^-1 ^produced T_4_/T_1 _= 0.4. However, at the same T_1_, T_4_/T_1 _was 0.78 in the subject who received the dose of 80 μg·kg^-1^.

When compared at identical values of T_1_, the T_4_/T_1 _ratios during the offset of NMB (lower panel in Figure 2) were lower than those observed during the onset. The ratios were similar among the different doses of vecuronium during the offset. Therefore, we have summarized the data for T_4_/T_1 _during the offset of NMB at T_1 _= 0.25, 0.5, 0.75, and 0.9, and present the mean ± SD in Figure 2.

**Figure 3:**
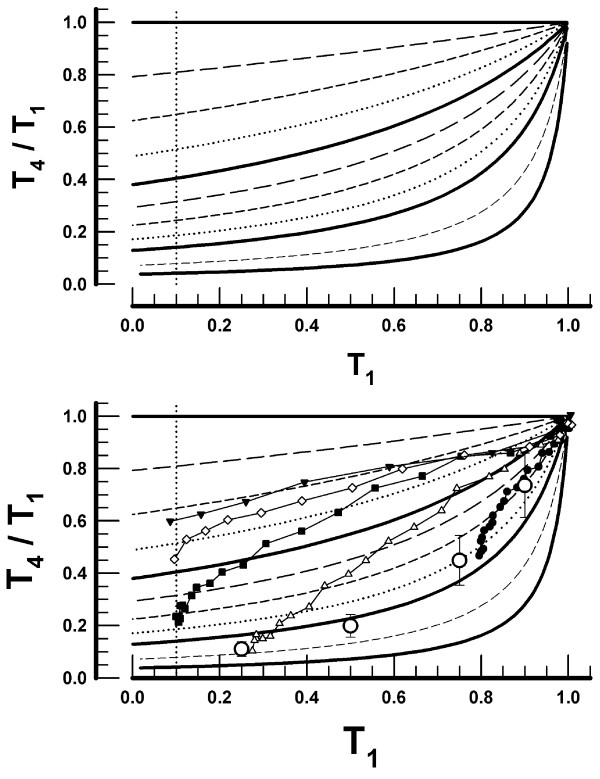
*Upper panel: *The ratios of the fourth to the first twitch (T_4_/T_1_) as a function of T_1_. The 11 curves were obtained in simulations assuming a constant amount of acetylcholine released by the first stimulus ([A]_1_) and either the same or smaller amounts released by the fourth stimulus ([A]_4_). The ratios of the initial concentrations of acetylcholine released by the fourth and the first stimulus are denoted by [A]_4_/[A]_1_. The continuous bold curves indicate, in descending order, [A]_4_/[A]_1 _ratios of 1.0, 0.9, 0.8, and 0.7, respectively. For the intermediate values of [A]_4_/[A]_1 _see Methods. *Lower panel: *The observed T_4_/T_1_-T_1 _data pairs during the onset of NMB and the mean values during the offset are inserted into the grid of the T_4_/T_1 _*versus *T_1 _curves presented in the upper panel. The observed data are those presented in Figure 2.

The simulations produced 11 curves, one for each [A]_4_/[A]_1 _value, relating T_4_/T_1 _to T_1 _(Figure 3). The curves indicate the inter-relationship between T_1_, T_4_/T_1_, and [A]_4_/[A]_1_. The relationship may be summarized as follows: (*i*) at a constant T_1_, the T_4_/T_1 _ratio is higher for higher [A]_4_/[A]_1_; (*ii*) at a constant [A]_4_/[A]_1_, the T_4_/T_1 _ratio is lower for lower values of T_1_; (*iii*) a given T_4_/T_1 _may be associated with many combinations of [A]_4_/[A]_1 _and T_1_; and (*iv*) a pair of T_4_/T_1_-T_1 _values is associated with a unique value of [A]_4_/[A]_1_. If the fourth stimulus releases the same number of ACh molecules as does the first, i.e. if [A]_4_/[A]_1 _= 1, then T_4 _remains equal to T_1_, i.e. T_4_/T_1 _= 1, for any value of T_1 _(Figure 3).

T_4_/T_1_-T_1 _data pairs observed during the onset of NMB produced by different doses of vecuronium, as well as the mean values of T_4_/T_1_-T_1 _observed in all patients during the offset of NMB (Figure 2), were superimposed on the grid relating T_4_/T_1 _ratios to T_1 _at different levels of [A]_4_/[A]_1 _(lower panel in Figure 3). The superimposition helps to clarify the relationship between the clinically observed data pairs and the simulated [A]_4_/[A]_1 _ratios.

The maximal decrease in [A]_4_/[A]_1 _appears to be influenced only minimally by the dose of vecuronium. For example, the smallest dose (15 μg·kg^-1^) decreased T_1 _to 0.8 and [A]_4_/[A]_1 _to 0.82. The largest dose (80 μg·kg^-1^) produced a complete ablation of T_1 _and yet decreased [A]_4_/[A]_1 _to only 0.7.

**Figure 4:**
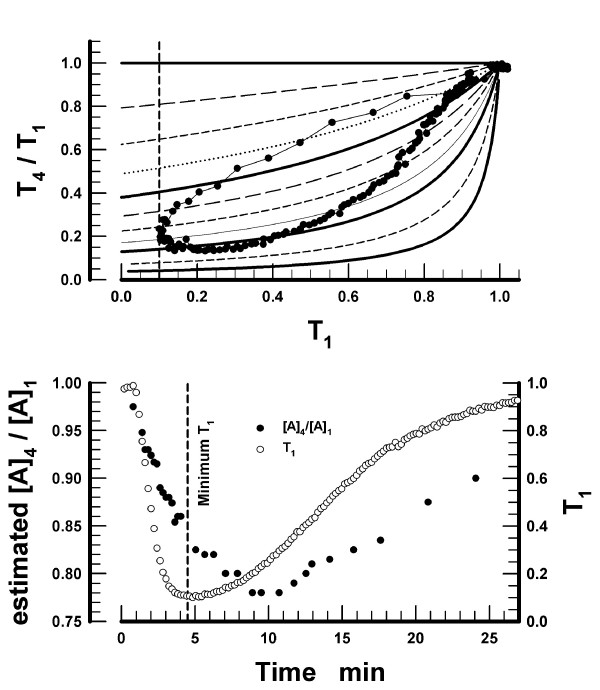
*Upper panel: *The complete set of the ratios of the fourth (T_4_) to the first twitch (T_1_) as a function of the concomitant T_1 _observed in a single subject. The dose of vecuronium was 25 μg·kg^-1^. The grid of the 11 curves is that presented in Figure 3. The dashed line parallel with the *Y*-axis indicates the minimal T_1_. *Lower panel: *The observed values of T_4_/T_1 _were interpolated between the [A]_4_/[A]_1 _curves to obtain [A]_4_/[A]_1 _values at times of the observed T_4_/T_1_-T_1 _data pairs. The estimated [A]_4_/[A]_1 _values are plotted as a function of time elapsed from the injection of vecuronium. The time-course of T_1 _depression in the same subject is also plotted (right *Y*-axis, filled circles). The dashed line parallel with the *Y*-axis indicates the time of the minimal T_1_.

The relationship between the observed T_4_/T_1_-T_1 _data pairs and the simulated T_4_/T_1 _*versus *T_1 _curves as a function of the [A]_4_/[A]_1 _ratios was examined in more detail in a single subject. The dose of vecuronium in this subject was 25 μg·kg^-1 ^and the complete observed time-course for T_4_/T_1 _*versus *T_1 _is presented (Figure 4). The results indicate that the T_4_/T_1 _ratios correspond to different [A]_4_/[A]_1 _during the onset and offset of NMB. Interpolation of the observed T_4_/T_1_-T_1 _data pairs between the simulated [A]_4_/[A]_1 _curves permitted us to plot the approximate [A]_4_/[A]_1 _ratios as a function of time after the injection of vecuronium in this subject (lower panel in Figure 4). The results reveal that, during the onset of NMB, the decrease in [A]_4_/[A]_1 _lags behind the decrease in T_1_. The peak depression of [A]_4_/[A]_1 _occurred approximately ten min after the injection of vecuronium, while the peak depression of T_1 _occurred after 4.5 min.

## Discussion

Our finding that vecuronium produces less fade for any given twitch depression during the onset compared to the offset of NMB (Figure 2) is consistent with the findings reported by other investigators [12-16]. We have also found that, during the onset and at similar T_1_, larger doses of vecuronium produce less fade than smaller doses (Figure 2). This finding is also consistent with the results from other investigations [14,15]. During the offset of NMB, the pooled T_4_/T_1_-T_1 _data pairs from all subjects (Figure 2) are similar to those reported by other investigators using vecuronium as well as other neuromuscular blocking drugs [12-16].

The primary purpose of the present study was to correlate twitch fade, as observed in the current study and by the other investigators, with the decrease in ACh release that would be necessary to produce the fade. The simulations are based on the postulate by Bowman [7] that twitch fade is due to the decreased amount of ACh released by the fourth stimulus as compared to that released by the first stimulus. The correlation between the clinically observed fade and the simulated decrease in [A]_4_/[A]_1 _allowed us to suggest tentative estimates for the vecuronium-induced decrease of ACh release elicited by the fourth stimulus during the TOF pattern of stimulation.

We have previously investigated in simulations twitch fade caused by nondepolarizing muscle relaxants [17]. However, in that report we were interested in the properties of the muscle relaxants in relation to their interaction with the presynaptic receptors (the affinities and the rates of interaction). In the current study, our focus is on the estimate of the magnitude of the [A]_4_/[A]_1 _ratio necessary to explain the fade.

The simulated result that no fade is present if the amount of ACh released by the fourth stimulus remains identical to that released by the first stimulus, i.e. if [A]_4 _= [A]_1 _(Figure 3), contradicts the suggestion that, even in the absence of neuromuscular blocking drugs, a spontaneous fall-off of the ACh quantal content occurs at 2 Hz stimulation frequency [18]. If there were a spontaneous fall-off in ACh quantal content upon repetitive stimulation, then in the presence of a drug that binds exclusively to the postsynaptic receptors, e.g. α-bungarotoxin or erabutoxin, the spontaneous fall-off in ACh release would, of necessity, lead to fade. However, it has been demonstrated repeatedly that these drugs produce a profound decrease in twitch height without producing fade [5,6,19]. Thus, the results of our simulations accord with, and support the notion of, no decline in ACh quantal content during the TOF pattern of stimulation in the absence of nondepolarizing neuromuscular blocking drugs [9,20,21].

The results from our simulations suggest that fade, i.e. T_4 _< T_1_, depends on both T_1 _and [A]_4_/[A]_1 _(Figure 3). Signal transmission across the neuromuscular junction results from a bimolecular reaction, i.e. from ACh binding to the postsynaptic receptors. A minor decrease in one of the partners, be it of ACh or the postsynaptic receptors, to its threshold concentration is not accompanied by failure of the muscle fibers to contract. This phenomenon is known as the safety factor [22]. Our simulations suggest that, with one partner at its threshold concentration, a concomitant decrease in the concentration of the other partner decreases the safety factor below its critical values in some muscle fibers. Failure of these fibers to contract leads to a decrease in twitch strength and, if the twitch is T_4_, a lower T_4_/T_1_.

All the T_4_/T_1_-T_1 _data pairs observed in our study and those reported by other investigators may be interpreted on the basis of the simulated T_4_/T_1 _*versus *T_1 _relationship and assuming different [A]_4_/[A]_1 _ratios. The observed T_4_/T_1 _ratios correspond to [A]_4_/[A]_1 _varying from 1.0 to 0.7. There appears to be a ceiling effect for the maximal decrease in [A]_4_/[A]_1_. Even the largest doses of vecuronium did not lead to [A]_4_/[A]_1 _< 0.7.

Whereas the lower [A]_4_/[A]_1 _ratios explain twitch fade, the lower ratios by themselves are insufficient to explain that: (*i*) the minimal value of T_4 _is attained later than the minimal value of T_1 _(Figure 1); (*ii*) twitch fade is not as extensive during the onset as during the offset of NMB (Figure 2); and (*iii*) during the onset, larger doses of vecuronium produce less fade at a given T_1 _than the smaller doses (Figure 2). These findings, confirming the previous findings of other investigators, are best explained by invoking a slow association of the nondepolarizing neuromuscular blocking drug with the presynaptic receptors and, hence, a slow development of the decrease in [A]_4_/[A]_1 _[8,14]. The slower development of the decrease in [A]_4_/[A]_1 _relative to the development of T_1 _depression (Figure 4) also explains the phenomenon that larger doses of neuromuscular blocking drugs produce less fade (during the onset of NMB) as compared with smaller doses. The reason is that the neuromuscular block develops faster with larger doses of a muscle relaxant. Thus, T_1 _= 0.05 is reached sooner following the doses 2 · ED95 or 3 · ED95 than following 1 · ED95 of the same muscle relaxant. The fast development of the decrease of T_1 _does not allow sufficient time for [A]_4_/[A]_1 _to decrease (*cf*. lower panel in Figure 4) and, as a consequence, the T_4_/T_1 _ratio is higher during the onset of the neuromuscular block following the larger doses of a muscle relaxant.

The correlation of the clinically observed time course of twitch fade to the simulated time course in the decrease in [A]_4_/[A]_1 _as well as the findings that the decrease in [A]_4_/[A]_1 _develops slowly do not reveal the processes leading to the decrease in [A]_4_/[A]_1_.

## Conclusion

The postulates that (*i*) the fourth stimulus releases fewer ACh molecules than the first, and (*ii*) the decrease in [A]_4_/[A]_1 _develops slower than the decrease in T_1_, adequately explain the T_4_/T_1_-*versus*-T_1 _relationship observed with vecuronium. The doses of vecuronium used in this study decrease the amount of ACh released by the fourth stimulus up to about 70% of the amount released by the first stimulus. However, the validity of these estimates is dependent on the validity of the assumptions made in simulations.

## Competing interests

The author(s) declare that they have no competing interests.

## Authors' contributions

SBB conceived the study, designed the clinical experiments, carried them out, performed data analysis, conceived the theoretical analysis and drafted the manuscript.

JK performed the clinical experiments and contributed in drafting the manuscript.

AA performed the calculations for the theoretical analysis part of the study and contributed to formulating the theoretical analysis and in drafting and revising the manuscript.

VN assisted in designing the experiments and in data analysis, assisted in calculations for the theoretical analysis, and contributed in drafting and revising the manuscript.

All authors read and approved the final manuscript.
